# Prevalence and risk factors for astigmatism in 7 to 19-year-old students in Xinjiang, China: a cross-sectional study

**DOI:** 10.1186/s12886-024-03382-0

**Published:** 2024-03-13

**Authors:** Yan Wang, Jingyu Mu, Yining Yang, Xiaolong Li, Han Qin, Batima Mulati, Zhen Wang, Wei Gong, Yong Zhao, Yunxian Gao

**Affiliations:** 1 Department of Ophthalmology, Traditional Chinese Medicine Hospital of Xinjiang Uyghur Autonomous Regional, No. 116 Huanghe Road, Shayibake District, 830099 Ürümqi, Xinjiang China; 2 School of Public Health, Xinjiang Medical University, No. 393 Xinyi Road, Xinshi District, Ürümqi, Xinjiang China; 3 Department of Ophthalmology, Shanghai General Hospital, Shanghai Jiao Tong University School of Medicine, No.100 Haining Road, Shanghai, China

**Keywords:** Astigmatism, Xinjiang, Children and adolescents, Ethnicity, Cross-sectional study

## Abstract

**Background:**

To investigate the prevalence and risk factors for astigmatism in 7-19-year-old students in Xinjiang, China.

**Methods:**

A school-based, cross-sectional study was conducted on students who underwent refraction examination in Xinjiang, China, between May and December 2019. The prevalence of astigmatism was determined. Astigmatism was defined as cylinder power (C) ≤-0.75 D, undefined astigmatism as ≤-1.50 D, and high astigmatism as C ≤-3.00 D. Astigmatism types were: against-the-rule astigmatism (maximum refraction of the main meridian in 180° ± 30°), with-the-rule astigmatism (maximum refraction of the main meridian at 90°±30°), and oblique astigmatism (all other cases).

**Results:**

Of the 71,838 students examined (51.0% boys, 7 − 19 years old), 25,945 (36.1%, 95%CI: 35.52−36.68%) had astigmatism and 1267 (1.8%, 95%CI: 1.07−2.53%) had high astigmatism. The prevalence of astigmatism was greater in Han individuals (39.6%) compared with the Hui (34.0%), Kazakh (34.0%), Kyrgyz (32.1%), and Uyghur (26.4%) populations. Among the 25,945 students with astigmatism, 19,947 had with-the-rule astigmatism (76.9%), 3405 had against-the-rule astigmatism (13.1%), and 2593 had oblique astigmatism (10.0%). Multivariable logistic regression analysis showed that ethnicity (Han individuals more susceptible), male gender, age, and refractive errors (myopia and hyperopia) were independently associated with astigmatism, high astigmatism, and with-the-rule astigmatism (all *P* < 0.05).

**Conclusions:**

The prevalence of astigmatism among children and adolescents in Xinjiang was 36.1%, including 1.8% of high astigmatism. In this population, astigmatism was mainly of the with-the-rule astigmatism type (76.9%). Han ethnicity, male gender, and myopia or hyperopia were independently associated with a high risk of astigmatism.

**Supplementary Information:**

The online version contains supplementary material available at 10.1186/s12886-024-03382-0.

## Background

Astigmatism is a refractive state in which the refractive power of the eye differs at different meridians, creating two focal lines and a minimal diffuse spot [1, 2]. In recent years, the rising annual global prevalence of astigmatism in children and adolescents has become an important clinical and public health concern. Uncorrected astigmatism significantly reduces visual function [1] and can significantly affect visual development in childhood, leading to amblyopia [2]. Previous studies reported a significant correlation between astigmatism and myopia development based on animal models and observations in longitudinal trials involving children [3, 4]. Therefore, early detection and treatment of astigmatism in children and adolescents is particularly important.

Currently, the pathogenesis of astigmatism in children and adolescents is unclear, and genetics, extraocular muscle tone, eyelid pressure, visual feedback, and environmental pollution have been implicated [5]. In addition, studies identified young age, severe refractive error (myopia or hyperopia), maternal smoking during pregnancy [6], eyelid flaps [7], early screen exposure [8, nystagmus [9], and environmental pollution [10] as factors associated with an increased risk of astigmatism.

Comprehensive analyses of astigmatism were never performed before in Xinjiang, China. Therefore, this study aimed to analyze the prevalence of, types of, and risk factors for astigmatism in children and adolescents living in Xinjiang, China.

## Methods

### Study design and participants

This cross-sectional study enrolled all students who underwent refraction examination by ophthalmologists from the Affiliated Hospital of Traditional Chinese Medicine of Xinjiang Medical University and the Xinjiang Uyghur Autonomous Region Academy of Traditional Chinese Medicine between May and December 2019. All refraction examinations were completed in the schools. A stratified cluster sampling method was designed based on schools in relevant geographical locations. These schools included Ürümqi, Tacheng, and Ili, located in Northern Xinjiang, and Kashi and Kizilsu, located in Southern Xinjiang.

The participant’s parents or guardians provided the signed informed consent form. The study followed the principles of the Declaration of Helsinki and was approved by the Ethics Committee of the Hospital of Traditional Chinese Medicine affiliated with Xinjiang Medical University. Students with significant ocular or systemic disease (e.g., cataract, glaucoma, ocular trauma, or trisomy 21), wearing orthokeratology lenses in the past three months before enrolment, or with incomplete information were excluded.

### Data collection and definitions

The data from 41 schools in the Xinjiang Region (China) were collected. The school provided detailed demographic and clinical data for each student, including name, gender, ethnicity, place of birth, date of birth, school name, grade level, and past medical history. All participants underwent an ophthalmic examination based on a standard protocol for common eye diseases by trained ophthalmic professionals (optometrists or ophthalmologists),, and refractive error measurement with a table-mounted TOPCON KR-8800 non-cycloplegic autorefractor, according to the “*The specification for screening of refractive error in primary and secondary school students (WS/T 663–2020)”.* Refractive error measurement was performed three times for each eye and averaged. If the difference between different readings of the same eye was greater than 0.5 D, the measurements were taken again. Ophthalmic examiners (ophthalmologists and optometrists, etc.) had been trained professionally. For quality control, the automatic optometer was calibrated daily before data collection, and about 5% of the examined students were randomly selected for repeated measurements.

Astigmatism was defined as cylinder power (C) ≤-0.75 D, undefined astigmatism as ≤-1.50 D, and high astigmatism as C ≤-3.00 D. The astigmatism types were against-the-rule astigmatism (maximum refraction of the main meridian in 180° ± 30°), with-the-rule astigmatism (maximum refraction of the main meridian at 90° ± 30°), and oblique astigmatism (all other cases). The average of three measurements was used for the angle. If the angle measurements were very different, the measurements were taken again. Spherical equivalent (SE) refraction was derived as SE = spherical power + 1/2 cylinder power. Myopia was classified as SE <-0.50 D and divided into low (-3.00 D to ≤-0.05 D), medium (-6.00 D to ≤-3.00 D), and high (≤-6.00 D) types. Hyperopia and emmetropia were defined as SE > + 0.50 D and − 0.50 D to ≤ + 0.50 D, respectively. After Pearson correlation analysis, there is a strong correlation between the binocular astigmatism of all subjects, with a correlation coefficient of 0.654 (*r* = 0.654, *p* < 0.001;95% CI: 0.650–0.658). Therefore, the data were obtained for the right eye.

### Statistical analysis

The sample size was estimated using the formula $n = \frac{{{u_\alpha }^2p(1 - p)}}{{{\delta ^2}}}$, considering myopia average annual incidence of *p* = 8%, α = 0.05, β = 0.20, δ = 0.1, *p* = 0.008, *n* = 1.962 × 0.08 × 0.9 ÷ 0.008^2^ ≈ 4418. The loss to follow-up rate was estimated as 10%, and the stratified cluster sampling efficiency was 1.5, *n* = 4418 ÷ 0.9 × 1.5 = 7362 people. The investigation was divided into primary and middle schools, and five ethnic groups were included. Therefore n = *N* × 2 × 5 ≈ 73,620 people, as shown in Supplemental Table 1.

SPSS 22.0 (SPSS, USA) was used for data analysis. The continuous data (e.g., age) were described as mean ± standard deviation and compared by analysis of variance (ANOVA). The categorical indicators (e.g., gender and ethnicity) were described as absolute logarithms and composition ratios and compared using the chi-square test (the corrected chi-square or Fisher’s exact probability test was used for R×C failure). Univariable analysis was performed to determine factors associated with astigmatism based on a logistic regression model, and parameters with *P* < 0.05 were included in the multivariable logistic regression model by the backward selection method. Odds ratios (ORs) and 95% confidence intervals (95%CIs) were determined. Two-sided *P* < 0.05 was considered statistically significant.

## Results

Twenty primary and 21 middle schools provided a list of 72,383 students; 547 students (0.75%) were excluded, of which 416 were beyond the age range of 7–19 years old, 34 could not cooperate with the examination or were absent during the examination, 32 had eye diseases, 35 had a history of eye surgery or eye trauma, and 30 had incomplete information. Finally, 71,838 children and adolescents aged 7–19 years (mean age, 11.59 ± 3.176) in Xinjiang, China, were analyzed. Table 1 presents the characteristics of the children and adolescents. Among them, 44.9% (*n* = 32,244) had − 0.50 D to < 0.50 D (Table 1).

**Table 1:** Characteristics of the study participants (*N* = 71,838)

Characteristics		n	Male n (%)	Female n (%)
Age (years)	7	6761	3386 (50.1)	3375 (49.9)
	8	7621	3987 (52.3)	3634 (47.7)
	9	7678	4002 (52.1)	3676 (47.9)
	10	7645	3963 (51.8)	3683 (48.2)
	11	8366	4347 (52.0)	4019 (48.0)
	12	7780	4115 (52.9)	3665 (47.1)
	13	5965	3107 (52.1)	2858 (47.9)
	14	5419	2824 (52.1)	2595 (47.9)
	15	4573	2404 (52.6)	2169 (47.4)
	16	3345	1562 (46.7)	1783 (53.3)
	17	3340	1423 (42.6)	1917 (57.4)
	18	2467	1115 (45.2)	1352 (54.8)
	19	878	372 (42.4)	506 (57.6)
Nationality	Han	45,021	23,571 (52.4)	21,450 (47.6)
	Uyghur	12,763	5914 (46.3)	6849 (53.7)
	Kazakh	1495	769 (51.4)	726 (48.6)
	Kyrgyz	5277	2639 (50.0)	2638 (50.0)
	Hui	5298	2735 (51.6)	2563 (48.4)
	Others	1984	979 (49.3)	1005 (50.7)
Education stage	Primary school	45,982	24,035 (52.3)	21,947 (47.7)
	Junior high school	16,068	8318 (51.8)	7750 (48.2)
	High school	9788	4254 (43.5)	5534 (56.5)
Spherical Equivalent (SE)	SE≤-6.00 D	1128	547 (48.5)	581 (51.5)
	-6.00 D < SE≤-5.00 D	1658	764 (46.1)	894 (53.9)
	-5.00 D < SE≤-4.00 D	2830	1313 (46.4)	1517 (53.6)
	-4.00 D < SE≤-3.00 D	3958	1887 (47.7)	2071 (52.3)
	-3.00 D < SE≤-2.00 D	5995	2828 (47.2)	3167 (52.8)
	-2.00 D < SE≤-0.50 D	19,937	9855 (49.4)	10,082 (50.6)
	-0.50 D < SE < 0.50 D	32,244	17,159 (53.2)	15,085 (46.8)
	0.50 D ≤ SE < 1.00 D	2715	1532 (56.4)	1183 (43.6)
	1.00 D ≤ SE	1373	722 (52.6)	651 (47.4)
	Total	71,838	36,605 (51.0)	35,231 (49.0)

**Fig. 1:**
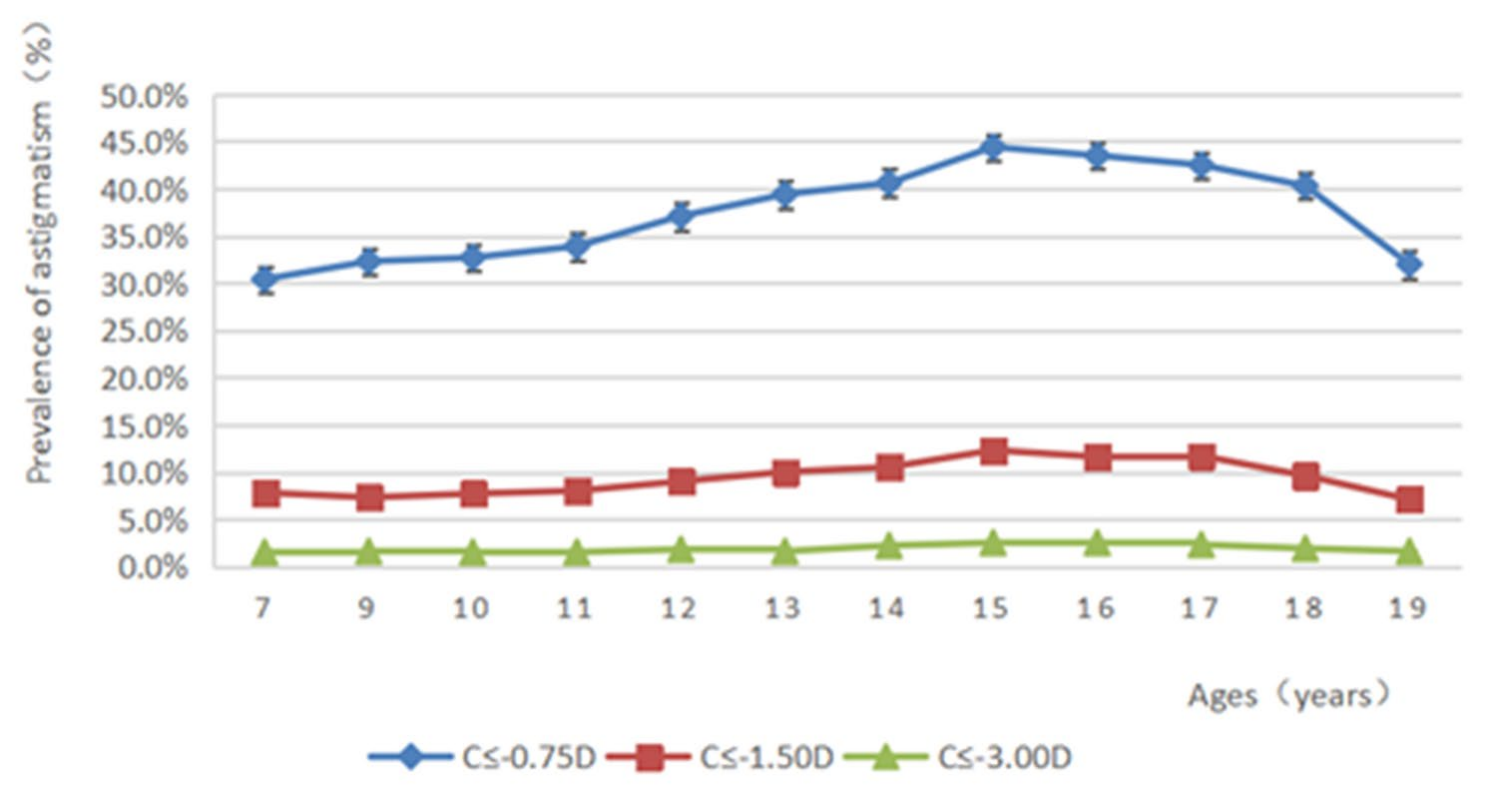
Prevalence rates of astigmatism (≥ 0.75 D and ≥ 1.5 D) and high astigmatism (≥ 3.0 D) by age from 7 to 19 years

**Fig. 2:**
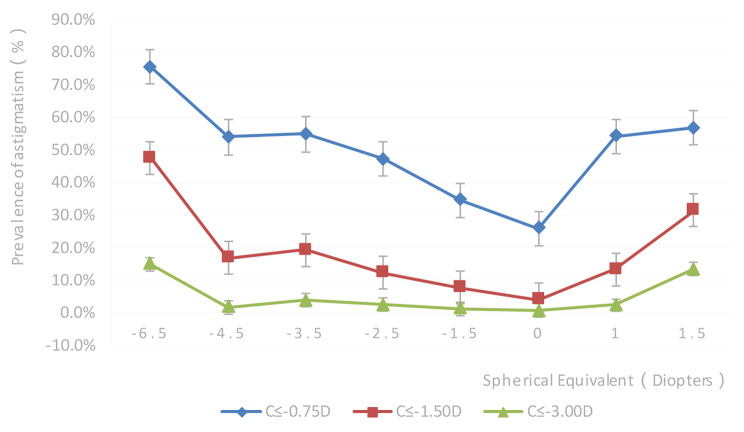
Prevalence rates of astigmatism (≥ 0.75 D and ≥ 1.5 D) and high astigmatism (≥ 3.0 D) by the magnitude of spherical equivalent

Of the 71,838 assessed students, 25,945 (36.1%, 95%CI: 35.52−36.68%, *P* < 0.001) had astigmatism ≤-0.75 D; 6465 (9.0%, 95%CI: 8.30−9.70%) and 1267 (1.8%, 95%CI: 1.07−2.53%, *P* < 0.001) had astigmatism ≤-1.50 D and ≤-3.00 D, respectively. There were statistically significant differences in astigmatism (≤-0.75 D) prevalence rates among different ages, genders, education levels, and ethnic groups (all *P* < 0.001). Astigmatism prevalence increased from 7 to 15 years, peaking at 15 years old. There was a decreasing trend from 15 to 19 years old (Fig. 1). SE refraction was also associated with astigmatism (≤-0.75 D, ≤-1.50 D, and ≤-3.00 D) (Table 2; Fig. 2). Astigmatism prevalence was significantly lower in primary school students (33.3%) compared with junior high (40.9%) and high (41.7%) school students (*P* < 0.05). Astigmatism prevalence was also higher in boys (37.8%) than in girls (34.3%) (*P* < 0.05). In addition, the astigmatism prevalence was higher in Han students (39.6%) compared with Uyghur (26.4%), Kazakh (34.0%), Kyrgyz (32.1%), and Hui (34.0%) students (*P* < 0.05).

**Table 2:** Prevalence rates of astigmatism by student characteristics (*N* = 71,838)

Characteristics		n	Astigmatism ≤-0.75 D, n (%)	Astigmatism ≤-1.50 D, n (%)	Astigmatism ≤-3.0 D, n (%)
Age (years)	7	6761	2056 (30.4)	526 (7.8)	99 (1.5)
	8	7621	2457 (32.2)	610 (8.0)	113 (1.5)
	9	7678	2481 (32.3)	563 (7.3)	122 (1.6)
	10	7645	2500 (32.7)	592 (7.7)	112 (1.5)
	11	8366	2833 (33.9)	669 (8.0)	126 (1.5)
	12	7780	2889 (37.1)	699 (9.0)	143 (1.8)
	13	5965	2350 (39.4)	597 (10.0)	95 (1.6)
	14	5419	2198 (40.6)	568 (10.5)	119 (2.2)
	15	4573	2029 (44.4)	564 (12.3)	113 (2.5)
	16	3345	1456 (43.5)	388 (11.6)	85 (2.5)
	17	3340	1421 (42.5)	390 (11.7)	78 (2.3)
	18	2467	994 (40.3)	237 (9.6)	48 (1.9)
	19	878	474 (32.0)	62 (7.1)	14 (1.6)
χ2			627.487	218.533	54.354
*P*-value			<0.001	<0.001	<0.001
Gender	Male	36,607	13,844 (37.8)	3646 (10.0)	696 (1.9)
	Female	35,231	12,101 (34.3)	2819 (8.0)	571 (1.6)
χ2			93.709	84.075	8.155
*P*-value			<0.001	<0.001	0.004
Nationality	Han	45,021	17,828 (39.6)	4846 (10.8)	878 (2.0)
	Uyghur	12,763	3368 (26.4)	579 (4.5)	136 (1.1)
	Kazakhs	1495	509 (34.0)	102 (6.8)	26 (1.7)
	Kyrgyz	5277	1695 (32.1)	306 (5.8)	98 (1.8)
	Hui	5298	1802 (34.0)	462 (8.7)	95 (1.8)
	Others	1984	743 (37.4)	170 (8.6)	24 (1.2)
χ2			811.136	557.162	51.008
*P*-value			<0.001	<0.001	<0.001
Education stage	Primary school	45,982	15,293 (33.3)	3672 (8.0)	707 (1.5)
	Junior high school	16,068	6567 (40.9)	1732 (10.8)	344 (2.1)
	High school	9788	4085 (41.7)	1061 (10.8)	216 (2.2)
χ2			454.042	160.324	37.858
*P*-value			<0.001	<0.001	<0.001
Spherical Equivalent (SE)	SE≤-6.00 D	1128	852 (75.5)	538 (47.7)	170 (15.1)
	-6.00 D < SE≤-5.00 D	1658	1047 (63.1)	262 (15.8)	49 (3.0)
	-5.00 D < SE≤-4.00 D	2830	1528 (54.0)	477 (16.9)	52 (1.8)
	-4.00 D < SE≤-3.00 D	3958	2173 (54.9)	767 (19.4)	151 (3.8)
	-3.00 D < SE≤-2.00 D	5995	2834 (47.3)	729 (12.2)	148 (2.5)
	-2.00 D < SE<-0.50 D	19,937	6913 (34.7)	1555 (7.8)	270 (1.4)
	-0.50 D ≤ SE ≤ + 0.50 D	32,244	8347 (25.9)	1340 (4.2)	179 (0.6)
	+ 0.50 D ≤ SE < + 1.00 D	2715	1471 (54.2)	364 (13.4)	64 (2.4)
	+ 1.00 D ≤ SE	1373	780 (56.8)	433 (31.5)	184 (13.4)
χ2			4724.538	4837.828	2649.796
*P*-value			<0.001	<0.001	<0.001

There were statistically significant differences in prevalence for astigmatism with ≤-1.50 D among different ages, genders, education levels, and ethnic groups (all *P* < 0.001) (Table 2). The prevalence rates of high astigmatism (C ≤-3.00 D) were significantly different among different ages, genders, education levels, and ethnic groups (all *P* < 0.05). The prevalence of high astigmatism tended to be stable in 7-13-year-old students, peaking at 2.5% at 15 and 16 years of age and showing a downward trend from 17 to 19 years (2.3%−1.6%). The prevalence of high astigmatism was significantly lower in primary school students (1.5%) than in junior high school students (2.1%) and high school students (2.2%). The prevalence of high astigmatism was higher in boys (1.9%) compared with girls (1.6%). The prevalence of high astigmatism was also higher in Han students (2.0%) compared with Uyghur (1.1%), Kazakh (1.7%), Kyrgyz (1.8%), and Hui (1.8%) counterparts. Taken together, these data suggested the prevalence of astigmatism (≤-0.75 D, ≤-1.50 D, and ≤-3.00 D) was correlated with age, male gender, and ethnicity (Table 2; Fig. 1).

Among students with astigmatism ≤-1.50 D, astigmatism prevalence rates were the highest in students with high myopia (≤-6.00 D 50.0%) and hyperopia ( > + 0.5 D, 21.5%) and the lowest in those with emmetropia (-3.00 D to <-0.50 D, 4.9%). Among students with astigmatism ≤-3.00 D, the astigmatism prevalence rates were the highest in students with high myopia (SE ≤-6.00 D, 15.6%) and hyperopia ( > + 0.5 D, 7.2%) and the lowest in those with emmetropia (-0.50 D to ≤ + 0.50 D, 0.8%) (Table 2). The trends of astigmatism prevalence (≤-0.75 D, ≤-1.50 D, and ≤-3.00 D) are shown in Fig. 1.

Among students with astigmatism ≤-0.75 D, those with high myopia (≤-6.00 D, 76.5%) and hyperopia ( > + 0.5 D, 56.3%) had the highest prevalence of astigmatism. The astigmatism prevalence was the lowest among students with emmetropia (-0.50 D to ≤ + 0.50 D, 28.8%). Multivariable logistic regression analysis showed that the risk of astigmatism ≤-0.75 D was significantly higher for students aged 8 − 19 years (*P* < 0.001) than for 7 years. Male students (*P* < 0.001) were more likely to have astigmatism than females. Compared with Uyghur students, Han (*P* < 0.001), Kazakh (*P* < 0.001), Hui (*P* < 0.001), and Kyrgyz (*P* < 0.001) students had an increased risk of astigmatism. The presence of refractive errors was also associated with astigmatism. Compared with emmetropia (-0.50 D ≤ SE ≤ + 0.50 D), hyperopia ( > + 0.5 D) and myopia (<-0.5 D) significantly increased the risk of astigmatism for hyperopia ( > + 0.5 D) (*P* < 0.001), for low myopia (-3.00 D to <-0.50 D) (*P* < 0.001), for medium myopia (-6.00 D to ≤-3.00 D) (*P* < 0.001), and high myopia (≤-6.0 D) (*P* < 0.001) (Table 3). Eleven or 15 − 17 years of age was independently associated with an increased risk of astigmatism ≤-1.50 D compared with the 7-year-old group (*P* = 0.012) for students aged 16. Male students were more likely to have astigmatism than females. Compared with Uyghur students, Han (*P* < 0.001), Kazakh (*P* < 0.001), Hui (*P* < 0.001), and Kyrgyz (*P* = 0.004) students had a higher risk of astigmatism. Compared with emmetropia cases (-0.50 D to ≤ + 0.50 D), hyperopia ( > + 0.5 D) and myopia (<-0.5 D) cases had significantly increased risk of astigmatism for hyperopia ( > + 0.5 D) (*P* < 0.001), low myopia (-3.00 D to <-0.50 D) (*P* < 0.001), medium myopia (-6.00 D to ≤-3.00 D) (*P* < 0.001), and high myopia (≤-6.00 D) (*P* < 0.001) (Table 4).

**Table 3:** Univariable and multivariable logistic regression analyses of risk factors for astigmatism ≥ 0.75 D (*N* = 25,945)

Characteristics	Astigmatism ≤-0.75 D (%)	Univariable logistic regression analysis		Multivariable logistic regression analysis	
		OR (95%CI)	*P*-Value	OR (95%CI)	*P*-Value
Age (year)			< 0.001		< 0.001
7	2056 (30.4)	Reference		Reference	
8	2457 (32.2)	1.089 (1.015–1.169)	0.018	1.135 (1.056–1.221)	0.001
9	2481 (32.3)	1.092 (1.018–1.172)	0.014	1.100 (1.023–1.182)	0.01
10	2500 (32.7)	1.112 (1.036–1.193)	0.003	1.074 (0.999–1.155)	0.053
11	2833 (33.9)	1.172 (1.094–1.255)	< 0.001	1.067 (0.993–1.146)	0.077
12	2889 (37.1)	1.352 (1.261–1.449)	< 0.001	1.202 (1.118–1.293)	< 0.001
13	2350 (39.4)	1.488 (1.382–1.601)	< 0.001	1.327 (1.228–1.434)	< 0.001
14	2198 (40.6)	1.562 (1.449–1.683)	< 0.001	1.326 (1.224–1.437)	< 0.001
15	2029 (44.4)	1.825 (1.688–1.973)	< 0.001	1.503 (1.383–1.634)	< 0.001
16	1456 (43.5)	1.764 (1.619–1.922)	< 0.001	1.585 (1.446–1.738)	< 0.001
17	1421 (42.5)	1.695 (1.555–1.847)	< 0.001	1.507 (1.374–1.653)	< 0.001
18	994 (40.3)	1.544 (1.403–1.699)	< 0.001	1.534 (1.385–1.699)	< 0.001
19	474 (32.0)	1.077 (0.926–1.253)	0.335	1.208 (1.030–1.417)	0.02
Gender					< 0.001
Female	12,101 (34.3)	Reference		Reference	
Male	13,844 (37.8)	1.162 (1.128–1.198)	< 0.001	1.186 (1.150–1.224)	< 0.001
Nationality			< 0.001		
Han	17,828 (39.6)	1.829 (1.751–1.910)	< 0.001	1.611 (1.536–1.690)	< 0.001
Uyghur	3368 (26.4)	Reference		Reference	
Kazakhs	509 (34.0)	1.440 (1.285–1.614)	< 0.001	1.415 (1.260–1.590)	< 0.001
Hui	1802 (34.0)	1.438 (1.342–1.541)	< 0.001	1.332 (1.239–1.433)	< 0.001
Kyrgyz	1695 (32.1)	1.320 (1.231–1.416)	< 0.001	1.271 (1.184–1.365)	< 0.001
Others	743 (37.4)	1.670 (1.513–1.844)	< 0.001	1.564 (1.413–1.732)	< 0.001
Spherical equivalent (right eye)			< 0.001		< 0.001
SE≤-6.00 D	773 (76.5)	8.216 (7.091–9.520)	< 0.001	6.391 (5.501–7.426)	< 0.001
-6.00 D < SE≤-3.00 D	4545 (57.1)	3.359 (3.196–3.531)	< 0.001	2.695 (2.551–2.848)	< 0.001
-3.00 D < SE<-0.50 D	8634 (36.7)	1.460 (1.410–1.512)	< 0.001	1.296 (1.248–1.346)	< 0.001
-0.50 D ≤ SE ≤ + 0.50 D	10,352 (28.4)	Reference		Reference	
SE > + 0.50 D	1641 (56.3)	3.250 (3.010–3.509)	< 0.001	3.437 (3.180–3.716)	< 0.001

CI: confidence interval; OR: odds ratio

**Table 4:** Univariable and multivariable logistic regression analyses of risk factors for astigmatism ≤-1.5 D (*N* = 6465)

Characteristics	Astigmatism ≤-1.5 D	Univariable logistic regression analysis		Multivariable logistic regression analysis	
		OR (95%CI)	*P*-Value	OR (95%CI)	*P*-Value
Age (year)			< 0.001		< 0.001
7	526 (7.8)	Reference		Reference	
8	610 (8.0)	1.031 (0.913–1.165)	0.619	1.119 (0.988–1.268)	0.076
9	563 (7.3)	0.938 (0.829–1.061)	0.31	0.959 (0.844–1.089)	0.52
10	592 (7.7)	0.995 (0.880–1.124)	0.935	0.943 (0.830–1.070)	0.362
11	669 (8.0)	1.030 (0.915–1.160)	0.623	0.883 (0.779–1.001)	0.052
12	699 (9.0)	1.170 (1.040–1.317)	0.009	0.958 (0.845–1.086)	0.505
13	597 (10.0)	1.318 (1.166–1.490)	< 0.001	1.061 (0.930–1.210)	0.38
14	568 (10.5)	1.388 (1.226–1.572)	< 0.001	1.013 (0.885–1.160)	0.846
15	564 (12.3)	1.668 (1.471–1.890)	< 0.001	1.169 (1.020–1.341)	0.025
16	388 (11.6)	1.555 (1.355–1.786)	< 0.001	1.212 (1.042–1.409)	0.012
17	390 (11.7)	1.567 (1.365–1.799)	< 0.001	1.172 (1.007–1.364)	0.04
18	237 (9.6)	1.260 (1.073–1.480)	0.005	1.096 (0.921–1.305)	0.302
19	62 (7.1)	0.901 (0.685–1.183)	0.453	0.920 (0.687–1.232)	0.575
Gender					< 0.001
Female	2819 (8.0)	Reference		Reference	
Male	3646 (10.0)	1.272 (1.208–1.339)	< 0.001	1.314 (1.246–1.386)	< 0.001
Nationality			< 0.001		
Han	4846 (10.8)	2.538 (2.323–2.773)	< 0.001	1.915 (1.741–2.106)	< 0.001
Uyghur	579 (4.5)	Reference		Reference	
Kazakhs	102 (6.8)	1.541 (1.239–1.915)	< 0.001	1.489 (1.193–1.859)	< 0.001
Hui	462 (8.7)	2.010 (1.771–2.282)	< 0.001	1.634 (1.431–1.865)	< 0.001
Kyrgyz	306 (5.8)	1.295 (1.123–1.494)	< 0.001	1.238 (1.071–1.431)	0.004
Others	170 (8.6)	1.972 (1.651–2.356)	< 0.001	1.672 (1.392–2.007)	< 0.001
Spherical equivalent (right eye)			< 0.001		< 0.001
SE≤-6.00 D	505 (50.0)	19.385 (16.985–22.125)	< 0.001	16.027 (13.931–18.437)	< 0.001
-6.00 D < SE≤-3.00 D	1409 (17.7)	4.174 (3.874–4.497)	< 0.001	3.511 (3.224–3.822)	< 0.001
-3.00 D < SE<-0.50 D	2139 (9.1)	1.938 (1.816–2.068)	< 0.001	1.753 (1.635–1.879)	< 0.001
-0.50 D ≤ SE ≤ + 0.50 D	1787 (4.9)	Reference		Reference	
SE > + 0.50 D	625 (21.5)	5.295 (4.789–5.855)	< 0.001	5.365 (4.844–5.942)	< 0.001

Male students had an increased risk of high astigmatism ≤-3.00 D compared with female students (*P* = 0.002). Compared with Uyghur students, Han (*P* = 0.001), Kazakh (*P* = 0.027), Hui (*P* = 0.015), and Kyrgyz (*P* < 0.001) students had elevated risk of high astigmatism. Compared with emmetropia (-0.50 D to ≤ + 0.50 D), hyperopia (SE > + 0.5 D) and myopia (SE <-0.5 D) had a significantly increased risk of high astigmatism (all *P* < 0.05) (Table 5).

**Table 5:** Univariable and Multivariable logistic regression analysis of risk factors for astigmatism ≥ 3.0 D (*N* = 1267)

Characteristics	Astigmatism ≤-3.0 D3.0 D	Univariable logistic regression analysis		Multivariable logistic regression analysis	
		OR (95%CI)	*P*-Value	OR (95%CI)	*P*-Value
Age (year)			< 0.001		0.297
7	99 (1.5)	Reference		Reference	
8	113 (1.5)	1.013 (0.772–1.329)	0.927	1.215 (0.922–1.602)	0.167
9	122 (1.6)	1.087 (0.832–1.419)	0.543	1.283 (0.976–1.686)	0.074
10	112 (1.5)	1.001 (0.762–1.314)	0.997	1.105 (0.835–1.463)	0.483
11	126 (1.5)	1.029 (0.789–1.341)	0.833	1.055 (0.800-1.392)	0.702
12	143 (1.8)	1.260 (0.973–1.631)	0.08	1.241 (0.945–1.630)	0.121
13	95 (1.6)	1.089 (0.820–1.446)	0.556	1.023 (0.758–1.381)	0.883
14	119 (2.2)	1.511 (1.155–1.977)	0.003	1.254 (0.938–1.675)	0.126
15	113 (2.5)	1.705 (1.298–2.239)	< 0.001	1.348 (1.004–1.809)	0.047
16	85 (2.5)	1.755 (1.309–2.352)	< 0.001	1.495 (1.091–2.048)	0.012
17	78 (2.3)	1.609 (1.192–2.171)	0.002	1.300 (0.941–1.796)	0.112
18	48 (1.9)	1.335 (0.943–1.891)	0.103	1.237 (0.856–1.788)	0.257
19	14 (1.6)	1.090 (0.620–1.917)	0.764	1.092 (0.608–1.960)	0.768
Gender					
Female	571 (1.6)	Reference		Reference	
Male	696 (1.9)	1.176 (1.052–1.315)	0.004	1.198 (1.069–1.342)	0.002
Nationality			< 0.001		0.002
Han	878 (2.0)	1.868 (1.558–2.240)	< 0.001	1.392 (1.146–1.692)	0.001
Uyghur	136 (1.1)	Reference		Reference	
Kazakhs	26 (1.7)	1.643 (1.077–2.508)	0.021	1.621 (1.056–2.488)	0.027
Hui	95 (1.8)	1.750 (1.347–2.273)	< 0.001	1.400 (1.068–1.834)	0.015
Kyrgyz	98 (1.8)	1.702 (1.307–2.216)	< 0.001	1.632 (1.249–2.133)	< 0.001
Others	24 (1.2)	1.137 (0.735–1.759)	0.565	0.952 (0.612–1.481)	0.827
Spherical equivalent (right eye)			< 0.001		< 0.001
SE≤-6.00 D	158 (15.6)	23.685 (19.273–29.108)	< 0.001	21.427 (17.048–26.930)	< 0.001
-6.00 D<SE≤-3.00 D	226 (2.8)	3.736 (3.131–4.457)	< 0.001	3.439 (2.824–4.187)	< 0.001
-3.00 D<SE<-0.50 D	391 (1.7)	2.158 (1.850–2.517)	< 0.001	2.063 (1.754–2.426)	< 0.001
-0.50 D ≤ SE ≤ + 0.50 D	283 (0.8)	Reference		Reference	
SE > + 0.50 D	209 (7.2)	9.872 (8.221–11.854)	< 0.001	10.128 (8.411–12.195)	< 0.001

Among the 25,945 students with astigmatism ≤-0.75 D, 19,947 had with-the-rule astigmatism (76.9%), 3405 had against-the-rule astigmatism (13.1%), and 2593 had oblique astigmatism (10.0%). There were statistically significant differences among ages, genders, ethnicities, education levels, refractive errors, and astigmatism types in 7–19 years (all *P* < 0.001). With increasing age, the prevalence of with-the-rule astigmatism in 7-15-year-old students showed an increasing trend (24.0−33.7%), while a decreasing trend (29.3%−18.8%) was found in 16-19-year-old students. The prevalence of with-the-rule astigmatism was higher in hyperopia (39.6%) and myopia (33.5%) than in emmetropia (21.7%). The prevalence of with-the-rule astigmatism was highest in Han students (32.5%), followed by Hui (27.8%), Kazakh (24.8%), Kyrgyz (17.0%), and Uyghur (15.6%) students (Table 6).

**Table 6:** Prevalence rates of different types of astigmatism (Astigmatism≤-0.75 D) (*N* = 71,838)

Characteristics		No astigmatism	With-the-rule	against-the-rule	Oblique	χ2	*P*-value
Age (year)	7	4705 (69.6)	1621 (24.0)	264 (3.9)	171 (2.5)	1000.100	< 0.001
	8	5164 (67.8)	2011 (26.4)	275 (3.6)	171 (2.2)		
	9	5197 (67.7)	1998 (26.0)	272 (3.5)	211 (2.7)		
	10	5145 (67.3)	1997 (26.1)	290 (3.8)	213 (2.8)		
	11	5533 (66.1)	2276 (27.2)	336 (4.0)	221 (2.6)		
	12	4891 (62.9)	2258 (29.0)	360 (4.6)	271 (3.5)		
	13	3615 (60.6)	1796 (30.1)	303 (5.1)	251 (4.2)		
	14	3221 (59.4)	1657 (30.6)	301 (5.6)	240 (4.4)		
	15	2544 (55.6)	1542 (33.7)	259 (5.7)	228 (5.0)		
	16	1889 (56.5)	981 (29.3)	255 (7.6)	220 (6.6)		
	17	1919 (57.5)	979 (29.3)	224 (6.7)	218 (6.5)		
	18	1473 (59.7)	666 (27.0)	197 (8.0)	131 (5.3)		
	19	597 (68.0)	165 (18.8)	69 (7.9)	47 (5.4)		
Gender	Male	22,763 (62.2)	10,843 (29.6)	1664 (4.5)	1337 (3.7)	132.507	< 0.001
	Female	23,130 (65.7)	9104 (25.8)	1741 (4.9)	1256 (3.6)		
Nationality	Han	27,193 (60.4)	14,635 (32.5)	1590 (3.5)	1603 (3.6)	2308.208	< 0.001
	Uyghur	9395 (73.6)	1985 (15.6)	902 (7.1)	481 (3.8)		
	Kazakhs	986 (66.0)	371 (24.8)	86 (5.8)	52 (3.5)		
	Kyrgyz	3582 (67.9)	897 (17.0)	570 (10.8)	228 (4.3)		
	Hui	3496 (66.0)	1474 (27.8)	175 (3.3)	153 (2.9)		
	Others	1241 (62.6)	585 (29.5)	82 (4.1)	76 (3.8)		
Education stage	Primary school	30,689 (66.7)	12,178 (26.5)	1818 (4.0)	1297 (2.8)	748.608	< 0.001
	Junior high school	9501 (59.1)	5004 (31.1)	867 (5.4)	696 (4.3)		
	High school	5703 (58.3)	2765 (28.2)	720 (7.4)	600 (6.1)		
Spherical equivalent (SE)	SE<-0.50 D	18,545 (57.1)	10,894 (33.5)	1631 (5.0)	1427 (4.4)	2215.31	< 0.001
	-0.50 D ≤ SE ≤ + 0.50 D	26,076 (71.6)	7898 (21.7)	1503 (4.1)	951 (2.6)		
	SE > + 0.50 D	1272 (43.7)	1155 (39.6)	271 (9.3)	266 (7.4)		
	Total	45,893 (63.9)	19,947 (27.8)	3405 (4.7)	2593 (3.6)		

Multivariable logistic regression analysis of risk factors for with-the-rule astigmatism (*n* = 19,947) showed similar results to astigmatism. Compared with 7-year-old children, 8-, 9- and 11-18-year-old students had elevated risk of with-the-rule astigmatism, with OR of 1.461 (95%CI: 1.334 − 1.600, *P* < 0.05) for 15-year-old students. Male students (OR = 1.211, 95% CI: 1.171–1.253, *P* < 0.001) had an elevated risk of with-the-rule astigmatism compared with female students. Compared to Uyghur students, Han (OR = 2.406, 95% CI: 2.275 − 2.545, *P* < 0.001), Kazakh (OR = 1.766, 95% CI: 1.550 − 2.012, *P* < 0.001), Hui (OR = 1.923, 95% CI: 1.774 − 2.084, *P* < 0.001), and Kyrgyz (OR = 1.134, 95% CI: 1.038 − 1.240, *P* = 0.005) students had increased risk of with-the-rule astigmatism. Compared with emmetropia cases (-0.50 D to ≤ + 0.50 D), hyperopia ( > + 0.5 D) and myopia (<-0.5 D) cases had significantly increased risk of with-the-rule astigmatism, with ORs of 3.206 (95% CI: 2.944 − 3.493, *P* < 0.001) for hyperopia (SE > + 0.5 D) and 1.516 (95% CI: 1.458 − 1.576, *P* < 0.001) for myopia (SE <-0.5 D) (Table 7).

**Table 7:** Multivariable logistic regression analysis of factors associated with astigmatism type among individuals with astigmatism ≤-0.75 D (*N* = 25,945)

Characteristics	With-the-Rule Astigmatism		Against-the-Rule Astigmatism		Oblique Astigmatism	
	OR (95%CI)	*P*-Value	OR (95%CI)	*P*-Value	OR (95%CI)	*P*-Value
Age (year)						
7	Reference		Reference		Reference	
8	1.155 (1.068–1.249)	< 0.001	1.025 (0.860–1.222)	0.779	0.990 (0.796–1.230)	0.925
9	1.089 (1.007–1.178)	0.032	0.989 (0.829–1.180)	0.902	1.176 (0.955–1.448)	0.127
10	1.067 (0.986–1.155)	0.106	1.046 (0.879–1.246)	0.61	1.169 (0.949–1.440)	0.143
11	1.090 (1.009–1.178)	0.029	1.099 (0.927–1.303)	0.276	1.095 (0.889–1.349)	0.392
12	1.220 (1.128–1.320)	< 0.001	1.310 (1.107–1.551)	0.002	1.510 (1.234–1.847)	< 0.001
13	1.384 (1.272–1.505)	< 0.001	1.410 (1.182–1.681)	< 0.001	1.928 (1.569–2.370)	< 0.001
14	1.425 (1.307–1.553)	< 0.001	1.484 (1.242–1.773)	< 0.001	2.017 (1.636–2.486)	< 0.001
15	1.667 (1.524–1.823)	< 0.001	1.553 (1.290–1.869)	< 0.001	2.374 (1.919–2.936)	< 0.001
16	1.635 (1.478–1.809)	< 0.001	1.959 (1.624–2.363)	< 0.001	3.267 (2.633–4.054)	< 0.001
17	1.633 (1.477–1.807)	< 0.001	1.761 (1.453–2.136)	< 0.001	3.251 (2.619–4.035)	< 0.001
18	1.644 (1.468–1.842)	< 0.001	1.945 (1.592–2.377)	< 0.001	2.685 (2.105–3.424)	< 0.001
19	1.194 (0.989–1.441)	0.065	1.602 (1.205–2.130)	0.001	2.495 (1.773–3.511)	< 0.001
Gender						
Female	Reference		Reference		Reference	
Male	1.211 (1.171–1.253)	< 0.001	1.030 (0.960–1.106)	0.411	1.131 (1.044–1.225)	0.003
Nationality						
Han	2.406 (2.275–2.545)	< 0.001	0.581 (0.529–0.638)	< 0.001	1.185 (1.056–1.330)	0.004
Uyghur	Reference		Reference		Reference	
Kazakhs	1.766 (1.550–2.012)	< 0.001	0.919 (0.728–1.160)	0.478	1.052 (0.782–1.415)	0.738
Hui	1.923 (1.774–2.084)	< 0.001	0.512 (0.431–0.607)	< 0.001	0.913 (0.753–1.106)	0.352
Kyrgyz	1.134 (1.038–1.240)	0.005	1.593 (1.422–1.784)	< 0.001	1.172 (0.995–1.381)	0.057
Others	2.159 (1.931–2.413)	< 0.001	0.669 (0.529–0.847)	0.001	1.216 (0.944–1.564)	0.13
Spherical equivalent (right eye)						
SE<-0.50 D	1.516 (1.458–1.576)	< 0.001	1.714 (1.580–1.859)	< 0.001	1.733 (1.577–1.904)	< 0.001
-0.50 D ≤ SE ≤ + 0.50 D	Reference		Reference		Reference	
SE > + 0.50 D	3.206 (2.944–3.493)	< 0.001	4.076 (3.528–4.709)	< 0.001	5.430 (4.621–6.379)	< 0.001

Aged 12-19-year was independently associated with an increased risk of against-the-rule astigmatism compared to 7-year-old students, with OR of 1.959 (95% CI: 1.624 − 2.363, *P* < 0.05) for 16-year-old students. Compared to the Uyghur ethnicity, Han (OR = 0.581, 95% CI: 0.529 − 0.638, *P* < 0.001) and Hui (OR = 0.512, 95% CI: 0.431 − 0.607, *P* < 0.001) were associated with lower risk of against-the-rule astigmatism; however, Kyrgyz (OR = 1.593, 95% CI: 1.422 − 1.784, *P* = 0.005) cases had a higher risk of astigmatism. Compared with emmetropia (-0.50 D to ≤ + 0.50 D), hyperopia ( > + 0.5 D) and myopia (<-0.5 D) had increased risk of against-the-rule astigmatism, with ORs of 4.076 (95% CI: 3.528 − 4.709, *P* < 0.001) for hyperopia (SE > + 0.5 D) and 1.714 (95% CI: 1.580 − 1.859, *P* < 0.001) for myopia (SE < -0.5 D) (Table 7).

Aged 12 − 19 years was also independently associated with an increased risk of oblique astigmatism compared to 7 years, with an OR of 3.267 (95% CI: 2.633 − 4.054, *P* < 0.001) for 16-year-old students. Male students (OR = 1.131, 95% CI: 1.044 − 1.225, *P* < 0.001) had an increased risk of oblique astigmatism compared with female students. Compared with the Uyghur ethnicity, Han (OR = 1.185, 95% CI: 1.056 − 1.330, *P* < 0.001) had an increased risk of oblique astigmatism. Compared with emmetropia cases (-0.50 D to ≤ + 0.50 D), hyperopia ( > + 0.5 D) and myopia (<-0.5 D) had increased risk of oblique astigmatism, with ORs of 5.430 (95% CI: 4.621 − 6.379, *P* < 0.001) for hyperopia ( > + 0.5 D) and 1.733 (95% CI: 1.577 − 1.904, *P* < 0.001) for myopia (<-0.5 D) (Table 7).

## Discussion

Comprehensive analyses of astigmatism were never performed before in Xinjiang, China. The present study showed a high prevalence of astigmatism among children and adolescents (7 − 19 years old) in Xinjiang (36.1%), including 1.8% of high astigmatism. In addition, with-the-rule astigmatism was the most abundant type in the examined students (76.9%). Moreover, Han ethnicity, male gender, age, and myopia or hyperopia were independent risk factors for astigmatism.

Although the study was performed in Xinjiang Province (25.9 million residents), the findings might provide a basis for managing astigmatism in Chinese children and adolescents. Although China is one of the most populous countries in the world, it is unsure whether the results could be applied to other populations (especially since the Han ethnicity is specific to China), and similar studies should be carried out in other countries. Nevertheless, the present study was mostly epidemiological and improved the knowledge related to astigmatism among Chinese children and adolescents. How such knowledge can translate into clinical improvements remains to be studied.

The current large-scale school survey found that the overall prevalence of astigmatism among children and adolescents aged 7 − 19 years in Xinjiang, China, was 36.1% (cylinder power ≤-0.75 D, non-cycloplegic autorefractor), including 9.0% individuals with cylinder power ≥ 1.50 D. The lack of diagnostic criteria for astigmatism results in significant differences in astigmatism prevalence among the available studies, making it impossible to analyze and compare the results directly. In a report assessing astigmatism in Yiwu, Zhejiang Province, China, the diagnostic criterion for astigmatism was ≥ 1.50 D (non-cycloplegic autorefractor). Compared with the present study, a higher prevalence of astigmatism was found in Yiwu City [11] compared with Xinjiang Province (14.2% vs. 9.0%). The discrepancy may be related to racial/ethnic differences. Results published by different studies worldwide regarding astigmatism prevalence are summarized in Table 8. Differences among studies can be due to several factors, including the tools used to screen for astigmatism, the definition used for astigmatism, the characteristics of the participants, the socioeconomic status, and the genetics of the populations. Indeed, the present study used the astigmatism definition commonly used in China: astigmatism was defined as C ≤-0.75 D, undefined astigmatism as ≤-1.50 D, and high astigmatism as C ≤-3.00 D. On the other hand, the American Academy of Ophthalmology uses the 3.00 D cutoff without distinctions in < 3.00 D [12]. The present study included individuals 7–19 years old, while other studies included slightly different age groups: 5–20 years old [11], 3–6 years old [13], 12 years old [14], and 6–14 years old [15]. Age is related to the progression of astigmatism, and including different age groups will lead to differences in epidemiological characteristics among studies [16, 17]. Xinjiang Province is located in Northwest China and is an area with a poorer socioeconomic status than in other parts of China and with less developed healthcare services. Finally, genetics are involved in the development of astigmatism, and loci specific to Asians and Europeans have been identified [18, 19]. Therefore, direct comparisons among studies cannot be performed because these factors are uncontrolled. International studies should be performed to examine these differences among multiple populations and by using the same definitions.

**Table 8:** Prevalence rates of astigmatism in different studies around the world

References	Country	n	Age (year)	Definition (D)	Refraction type	Prevalence (%)
In this study	China, Xinjiang	71,838	7–19	C≤-0.75	Non-cycloplegic autorefraction	36.10
				C≤-1.50	Non-cycloplegic autorefraction	9.00
Wang,2020 [11]	China, Yiwu	4801	5–20	C≤-1.50	Non-cycloplegic autorefraction	14.20
Li, 2019 [35]	China, Shanghai	7166	4–6	C≤-1.00	Non-cycloplegic autorefraction	12.70
Wang, 2014 [36]	China, Xuzhou	2255	2–6	C<-1.00	Cycloplegic autorefraction	8.80
Fan, 2011 [37]	China,HongKong	823	2–6	C≤-2.00	Cycloplegic autorefraction	5.70
SHIH, 2004 [22]	China, Taiwan	Year 1995, 11,175;	7–18	C≤-0.50	Non-cycloplegic autorefraction	Year 1995, 42.50
		Year 2000, 10,878				Year 2000, 51
				C≤-1.00	Non-cycloplegic autorefraction	Year 1995, 27.90;
						Year 2000, 32.60
Harrington, 2019 [38]	Ireland	1626	6–13	C≤-1.00	Non-cycloplegic autorefraction	19.20
Mayro, 2018 [39]	America, Philadelphia	18,974	Kindergarten to fifth grade	C<-1.00	Non-cycloplegic autorefraction	7.80
Hashemi, 2021 [23]	Iran, Shahroud	5528	6–12	C≤-0.75	Cycloplegic autorefraction	16.70
Norouzirad, 2015 [40]	Iran, Dezful	1375	6–15	C≤-0.50	Cycloplegic autorefraction	45.3
Fotouhi, 2011 [21]	Iran, Dezful	5544	14–18	C≤-0.75	Cycloplegic autorefraction	13.47
Chebil, 2015 [15]	Tunisian	6192	6–14	C≤-0.75	Non-cycloplegic autorefraction	6.67
Wajuihian, 2017 [41]	South African	1589	13–18	C≤-0.75	Non-cycloplegic autorefraction	3.10
Soler, 2015 [42]	Equatorial Guinea	425	6–16	C≤-0.75	Cycloplegic autorefraction	32.50

D: Diopter; NA: not available

In all age groups included in this study, the axial type of astigmatism was mainly with-the-rule astigmatism (75.9%), consistent with studies conducted in Yiwu City, China (85%) [11], Nanning, Guangxi Province, China (82.9%) [13] Anyang, Henan Province, China (58%) [14] and Tunisia (63.6%) [15], which all showed that with-the-rule astigmatism is the main type of astigmatism in children and adolescents. However, there were also different findings. A study of 3144 12-year-old children in 21 Australian schools found that against-the-rule astigmatism (42.2%) was the dominant type [20]. In addition, a survey of 5544 Iranian students also revealed against-the-rule astigmatism (48.14%) as the main type [21]. In a multivariable analysis of astigmatism type, age, gender, ethnicity, and refractive error were risk factors for astigmatism in children and adolescents aged 7–19 years in Xinjiang, China, and the risk of astigmatism at 15 years of age was relatively higher than that of 7-year-old children. Compared with girls, boys had a higher risk of astigmatism. Compared with Uyghur students, Han, Hui, and Kyrgyz students had a higher risk of astigmatism. Compared with emmetropia cases, myopia, and hyperopia cases had higher risk of astigmatism. The present study was cross-sectional and offers no insights about causality or the mechanisms of astigmatism. Longitudinal and mechanistic studies will be necessary. Still, a previous study reported that myopia or hyperopia was independently associated with astigmatism in Eastern China [11].

The prevalence of astigmatism generally varies with age. The results of this survey revealed that astigmatism prevalence increased with age from 7 − 15 years (30.4−44.4%), reached a peak at 15 years (44.4%), and decreased slightly from 15 − 19 years (43.5%−32.0%). It may be related to the small sample size for this age group. However, there was an increasing trend in astigmatism prevalence with increasing age and education level, corroborating previous studies in which the prevalence of astigmatism in children and adolescents tended to increase with age [15, 22, 23]. A survey in Yiwu City, China, also found that the higher the age and the school grade, the higher the prevalence of astigmatism [11]. However, studies performed in Iran [21] and Guangxi [13] showed no statistically significant difference in astigmatism prevalence based on age among children and adolescents. The differences arising from the above studies may be due to genetic and environmental factors related to the increased prevalence of astigmatism; alternatively, it may be that the higher the age, the higher the education level, and the more pronounced the effect of longer close-eye time on refractive status [1] and corneal changes [24].

This study also found that the prevalence of astigmatism was significantly higher in male students (37.8%) than in female students (34.3%; *P* < 0.001). In addition, multivariable regression analysis also showed that male gender was a risk factor for astigmatism. It was consistent with studies conducted in Yiwu City, China [11] and by the MEPEDS [25]. However, other reports suggested that astigmatism prevalence was significantly higher in female students than in their male counterparts [26, 27] or had no correlation with gender [15, 21]. In addition, in the above multivariable analysis, the female students were more likely to have astigmatism in the with-the-rule and oblique direction than male students, while there was no gender difference in against-the-rule astigmatism. These results indicated that the relationship between astigmatism prevalence and gender needs to be further explored by multicenter longitudinal studies.

The current study assessed differences in astigmatism prevalence among ethnic groups in the multi-ethnic Xinjiang region of China and found that ethnicity also played an important role. As shown above, astigmatism prevalence was significantly higher in the Han ethnicity (39.6%) compared with other ethnic minorities (26.4% of Uyghurs, 34.0% of Kazakhs, 32.1% of Kyrgyz, and 34.0% of Hui). In addition, multivariable analysis showed higher astigmatism risk in Han, Kazakh, Kyrgyz, and Hui students compared with Uyghur students. The conclusions of this study were consistent with those of other multi-ethnic areas in China, in which astigmatism prevalence in Yunnan, China, was higher in Han individuals than in the Yi population (60.07% vs. 50.67%) [28]. It was also found that astigmatism prevalence was higher in Han individuals than in the Tibetan ethnicity (72.14% vs. 64.94%) in the multi-ethnic area of Qinghai, China [29]. Scholars have found elevated astigmatism prevalence in Asians compared with other races [30, 31]. The MEPEDS et al. also found that Hispanics had higher astigmatism rates than African Americans and Caucasians [5, 25, 30]. These results suggest that the astigmatism detection rate may be related to genetic differences in diagnostic criteria, measurement method, region, education level, lifestyle, and race.

This study also found that refractive error was strongly associated with astigmatism prevalence and type. Students with hyperopia ( > + 0.5 D) were 3.437 times more likely to develop astigmatism than those with emmetropia (-0.50 D to ≤ + 0.50 D), while individuals with low myopia (-3.00 D to <-0.50 D), medium myopia (-6.00 D to ≤-3.00 D) and high myopia (SE ≤-6.00 D) were 1.296 times, 2.695 times and 6.391 times more likely to develop astigmatism, respectively, compared with emmetropia cases. This corroborated previous studies conducted in Yiwu, China [32] and Anyang, China [14], as well as a study conducted by the MEPEDS [25] and the US VIP multicenter study [6]. We can conclude that children with refractive errors are more likely to develop astigmatism than those without refractive errors. However, because our and most previous studies had a cross-sectional design, we could not determine the causal relationship, and further longitudinal studies would better assess the association between refractive status and astigmatism prevalence. The present investigation also found that the type of astigmatism was correlated with the degree of refractive error, with hyperopia ( > + 0.5 D) cases being 3.206 times more likely to have with-the-rule astigmatism than emmetropia (-0.50 D to ≤ + 0.50 D) cases, while myopic students (SE < -0.5 D) were 1.516 times more likely to have with-the-rule astigmatism than emmetropia students. It was consistent with findings by the US VIP multicenter study [6]. Not only was refractive error associated with with-the-rule astigmatism, but spherical and cylinder powers were also shown to have significant and independent effects. A survey of 90,884 individuals aged 21 − 40 years in northern England found that an increase in spherical or cylinder power in astigmatic individuals increases their odds of developing with-the-rule astigmatism and also detected more significant oblique astigmatism in myopic individuals [33]. Other studies have also found correlations between high spherical powers and with-the-rule astigmatism, with against-the-rule astigmatism increasing with decreasing spherical power [34]. Studies in Taiwan and Iran [22, 23] also confirmed the relationship between astigmatic axis position and spherical refractive error. In the above multivariable analysis, we also found that myopic or hyperopic individuals were more likely to develop astigmatism in the against-the-rule and oblique astigmatism than in emmetropia. Further longitudinal studies are needed to evaluate the causal relationship between the variation of the astigmatic axis and the degree of refractive error.

The strengths of this study are as follows. First, the sample size was large, including many regions and representative ethnicities. Secondly, there are relatively few reports on astigmatism prevalence, especially in Western China, a gap closed by this study. In addition, data for several diagnostic criteria were provided in this study, which could be compared with other investigations. However, there were also some limitations in this study. First, non-cycloplegic autorefraction reduces the accuracy of the diopter number. Secondly, it was a cross-sectional study, which cannot determine the causal relationships, e.g., between age and refractive state and between astigmatism prevalence and astigmatic axis, in children and adolescents. Further longitudinal cohort studies are required to accurately and scientifically analyze astigmatism data and provide an effective scientific basis for the prevention and control of astigmatism.

## Conclusions

The above large-scale school survey showed that astigmatism was relatively high among children and adolescents in Xinjiang, China, with astigmatism mainly being with-the-rule astigmatism, which increased with age and education level. The risk of astigmatism, high astigmatism, and with-the-rule astigmatism was increased in the Han ethnicity, males, and myopia or hyperopia cases. The possible causal relationships of refractive error (myopia or hyperopia), ethnicity, astigmatism, and the astigmatic axis must be further confirmed by multicenter longitudinal studies with large sample sizes.

### Electronic supplementary material

Below is the link to the electronic supplementary material.

Supplementary Material 1

## Data Availability

All data generated or analyzed during this study are included in this published article and its supplementary information files.

## Abbreviations

SE: Spherical equivalent refraction
C: Cylinder power
D: Diopter
NA: Not available
MEPEDS: Multi-Ethnic Pediatric Eye Disease Study
CI: Confidence Interval
VIP: Vision in Preschoolers
