# Supplementary material for: Prevalence and risk factors for astigmatism in 7 to 19-year-old students in Xinjiang, China: a cross-sectional study
Source: BMC Ophthalmol. 2024 Mar 13;24:116. doi: 10.1186/s12886-024-03382-0 (PMC10935971; doi:10.1186/s12886-024-03382-0)
Supplement: Supplementary file 1 — Supplementary Material 1 [file 12886_2024_3382_MOESM1_ESM.docx]

**Prevalence and risk factors for astigmatism in 7 to 19-year-old students in Xinjiang, China: A cross-sectional study**

**Running title:** Prevalence and risks for astigmatism

Yan Wang^#, 1^, Jingyu Mu^#, 1^, Yining Yang^1^, Xiaolong Li^1^, Han Qin^1^, Batima·Mulati^1^, Zhen Wang^2^, Wei Gong^3^, Yong Zhao^*,^ ^1^, Yunxian Gao^*, 1^

^1^ Department of Ophthalmology, Traditional Chinese Medicine Hospital of Xinjiang Uyghur Autonomous Region, NO. 116 Huanghe Road, Shayibake District, Ürümqi, Xinjiang, China.

^2^ School of Public Health, Xinjiang Medical University, NO. 393 Xinyi Road, Xinshi District, Ürümqi, Xinjiang, China.

^3^ Department of Ophthalmology, Shanghai General Hospital, Shanghai Jiao Tong University School of Medicine, No.100 Haining Road, Shanghai, China

^#^YW and JYM contributed equally to the work presented here and should therefore be regarded as equivalent authors.

**^*^Corresponding Authors**

Yong Zhao

Address: NO. 116 Huanghe Road, Shayibake District, Ürümqi, Xinjiang 830099, China.

E-mail: 13999972362@163.com

Yunxian Gao

Address: NO. 116 Huanghe Road, Shayibake District, Ürümqi, Xinjiang 830099, China.

Tel: +86-18609915348

E-mail: gaoyx6362@163.com

**Supplemental Table 1: Sampling scheme**

| Region | The number of sampling | School | Total enrollment of students | Nationality | Number of individual ethnic groups |
| --- | --- | --- | --- | --- | --- |
| Urumqi City | 51534 | elementary school | 25767 | Uyghurs | 24324 |
|  |  |  |  | Han Chinese | 18449 |
|  |  |  |  | Ha people | 4793 |
|  |  |  |  | Hui | 2371 |
|  |  |  |  | other | 1598 |
|  |  | secondary school | 25767 | Uyghurs | 24324 |
|  |  |  |  | Han Chinese | 18449 |
|  |  |  |  | Ha people | 4793 |
|  |  |  |  | Hui | 2371 |
|  |  |  |  | other | 1598 |
| Croatia | 11043 | elementary school | 7412 | Uyghurs | 3498 |
|  |  |  |  | Han Chinese | 2653 |
|  |  |  |  | Ha people | 689 |
|  |  |  |  | Hui | 341 |
|  |  |  |  | other | 230 |
|  |  | secondary school | 3632 | Uyghurs | 1714 |
|  |  |  |  | Han Chinese | 1300 |
|  |  |  |  | Ha people | 338 |
|  |  |  |  | Hui | 167 |
|  |  |  |  | other | 113 |
| Tacheng/Kashgar | 3681 | elementary school | 553 | Uyghurs | 261 |
|  |  |  |  | Han Chinese | 198 |
|  |  |  |  | Ha people | 51 |
|  |  |  |  | Hui | 25 |
|  |  |  |  | other | 17 |
|  |  | secondary school | 3129 | Uyghurs | 1477 |
|  |  |  |  | Han Chinese | 1120 |
|  |  |  |  | Ha people | 291 |
|  |  |  |  | Hui | 144 |
|  |  |  |  | other | 97 |
| Ili Kazakh Autonomous Prefecture | 7362 | elementary school | 1474 | Uyghurs | 696 |
|  |  |  |  | Han Chinese | 528 |
|  |  |  |  | Ha people | 137 |
|  |  |  |  | Hui | 68 |
|  |  |  |  | other | 46 |
|  |  | secondary school | 5888 | Uyghurs | 2779 |
|  |  |  |  | Han Chinese | 2108 |
|  |  |  |  | Ha people | 548 |
|  |  |  |  | Hui | 271 |
|  |  |  |  | other | 183 |
